# Laterality of anterior temporal lobe repetitive transcranial magnetic stimulation determines the degree of disruption in picture naming

**DOI:** 10.1007/s00429-017-1430-2

**Published:** 2017-07-29

**Authors:** Anna M. Woollams, Lee J. Lindley, Gorana Pobric, Paul Hoffman

**Affiliations:** 1 Neuroscience and Aphasia Research Unit, Division of Neuroscience and Experimental Psychology, University of Manchester, Manchester, M13 9PL United Kingdom; 2 Centre for Cognitive Ageing and Cognitive Epidemiology (CCACE), Department of Psychology, University of Edinburgh, Edinburgh, EH8 9JZ United Kingdom

**Keywords:** Semantics, Naming, Anterior temporal lobes, Laterality, Speech production, Connectivity

## Abstract

The bilateral anterior temporal lobes play a key role in semantic representation. This is clearly demonstrated by the performance of patients with semantic dementia, a disorder characterised by a progressive and selective decline in semantic memory over all modalities as a result of anterior temporal atrophy. Although all patients exhibit a progressive decline in both single-word production and comprehension, those with greater atrophy to the left anterior temporal lobe show a stronger decline in word production than comprehension. This asymmetry has been attributed to the greater connectivity of the left anterior temporal lobe with left-lateralised speech production mechanisms. Virtual lesioning of the left ATL using offline repetitive transcranial magnetic stimulation (rTMS) has been shown to disrupt picture naming, but, the impact of right ATL rTMS is yet to be explored. We tested the prediction that disruption of picture naming in normal participants by rTMS should be greater for the left than the right ATL. We found a significant increase in picture naming latencies specifically for stimulation of the left ATL only. Neither left nor right ATL TMS slowed performance in a number naming control task. These results support the hypothesis that although both temporal lobes are part of a widespread semantic network in the human brain, the left anterior temporal lobe possesses a stronger connection to left-lateralised speech production areas than the right temporal lobe.

## Introduction

Converging evidence from multiple methodologies indicates that the bilateral anterior temporal lobes (ATL) play an important role in representing semantic knowledge. The most well-known source of evidence for this view is the syndrome of semantic dementia, in which bilateral ATL atrophy is associated with a selective and eventually profound deterioration in verbal and non-verbal semantic knowledge (Patterson et al. 2007; Bozeat et al. 2000; Snowden et al. 1989). Similar, albeit less severe, deficits are found in patients with unilateral surgical resection of the ATL (Lambon Ralph et al. 2012; Wilkins and Moscovitch 1978; Antonucci et al. 2008) and ATL activation has been observed during semantic processing using intracranial recordings (Shimotake et al. 2015; Nobre et al. 1994), MEG (Marinkovic et al. 2003) and in a range of functional neuroimaging studies (Visser et al. 2010; Humphreys et al. 2015; Vandenberghe et al. 1996). Evidence indicates that both left and right ATLs make important contributions to semantic processing. For example, left and right ATL resections both result in semantic deficits of similar levels of severity (Lambon Ralph et al. 2012). Similarly, semantic tasks commonly elicit bilateral ATL activation in functional neuroimaging studies, though often more prominently in the left hemisphere (Rice et al. 2015b).

Though it is clear that both ATLs contribute to semantic knowledge, the degree of functional specialization across hemispheres is an important and unresolved question. Gainotti and colleagues have proposed that the left ATL is specialised for the representation of verbal semantic knowledge and the right for non-verbal information (Gainotti 2012, 2014; Gainotti et al. 2003). This modality view is supported by some studies of semantic dementia patients, in whom atrophy is often asymmetric, disproportionately affecting either the right or (more often) left ATL (Hodges et al. 2010). Snowden et al. (2004), for example, found that semantic dementia patients with left-dominant damage performed more poorly on recognition of famous people when the stimuli were presented as written names, rather than pictures. The reverse was true for right-dominant cases (see Gainotti 2012, for a more detailed review). Functional neuroimaging studies in healthy individuals provide less support for this view, however. In a recent meta-analysis of 97 functional neuroimaging studies, Rice et al. (2015b) found that the majority of studies reported activation in both ATLs, irrespective of whether information was presented verbally or non-verbally. Among studies reporting unilateral activity, modality of the stimulus had no effect on whether activation was found in the left or right ATL. Of course, functional neuroimaging and lesion studies have rather different strengths and weaknesses and the reason for the divergence of evidence on this issue is not entirely clear. In any case, the present study was designed to test a different, although not mutually exclusive, possibility: that the left ATL exhibits specialization for semantic tasks requiring speech production.

Speech production, which we define simply as the act of outputting a sequence of spoken phonemes, is the paradigmatic example of a left-lateralised function (Pascual-Leone et al. 1991). It is well established that semantic dementia patients with left-dominant ATL atrophy are markedly more anomic than those with right-lateralised damage, even when equating for the severity of their receptive semantic deficits (Lambon Ralph et al. 2001). Similar effects have been observed in patients with ATL damage arising from unilateral resection (Lambon Ralph et al. 2012; Drane et al. 2008, 2013) and other aetiologies (Acres et al. 2009; Lambon Ralph et al. 2010; Damasio et al. 2004; Patterson et al. 2015). Functional neuroimaging studies of semantic tasks that involve speech production also produce more left-lateralised pattern of ATL activation more than those that use receptive tasks (Rice et al. 2015b). The left ATL, therefore, appears to play a more centralized role in phonological output based on semantic knowledge.

Computational models have simulated such findings by assuming that both ATLs are equally involved in representing semantic information but that the left ATL has stronger connections to left-lateralised speech production systems (Lambon Ralph et al. 2001; Schapiro et al. 2013; Rice et al. 2015a). According to this connectivity view, damage to the left ATL, therefore, has a more significant effect on the mapping from semantics to speech output. This position is supported by known asymmetries in white matter connectivity: the uncinate fasciculus that connects the anterior temporal lobe to the left inferior frontal gyrus has been reported to be of a higher volume in the left than right hemisphere (Leng et al. 2016; Parker et al. 2005), and the connections of the arcuate fasciculus linking anterior temporal lobe to the left inferior frontal gyrus has been shown to have higher consistency in the left than right hemisphere (Papinutto et al. 2016).

In the Lambon Ralph et al. (2001) and Schapiro et al. (2013) models, distributed semantic representations interact directly with representations of output phonology, without the need for an intermediate lexical level of representation. Other models take a different view, proposing that lexical representations in the ATL link semantic representations stored elsewhere with phonological information (Damasio et al. 2004; Drane et al. 2013). Drane et al. (2013), for example, suggested that the left ATL is specialised for lexical-semantic access while the right ATL is involved in visual-semantic analysis. On this view, semantic representations are stored out with the left ATL, but this region plays a critical role in linking semantic knowledge with the phonological system. This hypothesis was motivated by data from patients with temporal lobe epilepsy when naming and recognising famous faces, though it is assumed to apply to object concepts more generally. Although the details of these models vary, they share the core assumption that the left ATL is more closely involved than the right in semantically-driven word retrieval tasks.

Most evidence for ATL specialization comes from neuropsychological and functional neuroimaging studies. Transcranial magnetic stimulation (TMS) offers an important complementary approach. Unlike functional neuroimaging, TMS permits the establishment of causative relationships between brain function and behavioural performance (Walsh and Cowey 2000). Unlike patient lesion studies, the “virtual lesions” induced by TMS are focal and their location is under precise experimental control. In addition, neural disruption is temporary and takes place in healthy individuals, thus avoiding complications arising from functional reorganization in patients with chronic disorders. This can present a particular issue in patients with ATL resections, who have typically experienced chronic temporal lobe epilepsy from an early age (Powell et al. 2007).

A number of studies have shown that TMS applied to either the left or right lateral ATLs disrupts performance on a variety of semantic tasks (Pobric et al. 2010a; Lambon Ralph et al. 2009; Pobric et al. 2007; Hoffman and Crutch 2016). Importantly, few studies have explored differential effects of left vs. right ATL stimulation. Pobric et al. (2010a) found that stimulation to either ATL slowed performance on two semantic association tasks—one using words and one pictures—but with no significant differences in effects across the two hemispheres. Bonnì et al. (2015) used the same tasks with continuous theta-burst stimulation and found paradoxically that stimulation improved performance, but only for the picture task and only with right ATL stimulation. Neither study probed speech production. Previous reports have established that virtual lesioning of the left ATL disrupts picture naming performance (Pobric et al. 2010b). This disruption is particularly pronounced for items that are atypical of their semantic category (e.g., peguin for bird) (Woollams 2012), and when participants are required to name items at the subordinate level (e.g., labrador for dog) (Pobric et al. 2007). Yet the impact of a virtual lesion of the right ATL upon picture naming has never been investigated. In the present study, we compared the effects of left vs. right ATL TMS on a picture-naming task and on a matched receptive semantic task (spoken word-to-picture matching), using parietally mediated (Butterworth et al. 2001) number naming and number matching tasks to control for any general effects of stimulation. We tested the prediction of the connectivity view of ATL specialization that naming performance would be disproportionately affected by stimulation of the left ATL, whereas performance during spoken word-to-picture matching would not be sensitive to the laterality of ATL stimulation.

## Method

### Design

The present study utilized rTMS using the virtual lesion method in which, after baseline behavioural assessment, a train of rTMS is delivered offline (without a concurrent behavioural task) and then behavioural performance is investigated again during the temporary refractory period induced by the TMS. Performance before and after left and right ATL TMS in the semantic tasks of interest—picture naming and spoken word-to-picture matching—was compared to number naming and number matching tasks. These control for the input and output requirements of the semantic tasks, but the use of numbers as stimuli meant that no disruption of performance from left or right ATL TMS was expected, as number processing is mediated by parietal regions (Butterworth et al. 2001) and is well-preserved in SD (Jefferies et al. 2004).

The full study design, therefore, involved a 2 (modality: semantic vs. numeric) by 2 (task: naming vs matching) by 2 (stimulation: pre-TMS vs. post-TMS) by 2 (laterality: left ATL vs. right ATL) fully within participants design. Each participant attended a first session where they completed tasks involving producing and matching both object pictures and number names, with the order of these four tasks counterbalanced in order of enlistment. They then received 10 min of 1 Hz rTMS to the ATL, with the side of stimulation alternating over participants. They then completed the four tasks again, in the same order as prior to stimulation but with new items. Participants then returned for a second session after at least 2 weeks where they underwent the same task sequence, but with the assignment of item sets to pre- and post-TMS reversed and the opposite side of ATL stimulation.

### Participants

Twelve right-handed participants took part in the study (7 females). All participants were native English speakers and right handed, with a laterality quotient of at least +80 on the Edinburgh Handedness Inventory (Oldfield 1971) ($\bar{x}$ = 91.36; $\sigma_{x}$ = 7.45). Additionally all participants were free from any history of neurological disorder or mental illness, and none were currently taking any medication. All had normal or corrected-to-normal vision. Participants gave written informed consent and the experimental procedure was reviewed and approved by the University of Manchester Research Ethics Committee. Participants were reimbursed for their participation.

### Stimuli

For each of the naming and matching tasks, 80 picture stimuli and 40 number stimuli were used (160 picture and 80 number stimuli in total—please see Appendix for list). The picture stimuli were drawn from the International Picture Naming Project database (Szekely et al. 2004) which contains images taken from the original Snodgrass and Vandewart picture set, the Boston Naming Test and Peabody Picture Vocabulary test amongst others. Pictures were drawn across a number of different categories, such as birds, animals, fruit, household items, tools and vehicles. The pictures used were selected based upon specific inclusion criteria, with all items having a greater than 85% name agreement and a frequency of less than 200 occurrences per million (as assessed by the MCWord database; Medler and Binder 2005). The number naming and matching tasks involved English names for six-digit numbers (e.g. 238,966, “two hundred and thirty-eight thousand nine hundred and sixty-six”), as pilot studies found that these longer numbers provided similar naming and matching latencies to those for the picture items. Each group of items for naming and matching was split into two sets matched for name agreement, word frequency and response latency as identified in our pilot studies, with one set used in the pre rTMS baseline condition and the other immediately after the application of rTMS in each session. The two sets were counterbalanced across participants.

### Procedure

A PC running DMDX (Forster and Forster 2003) presented the stimuli and recorded the reaction times of participants’ responses. The participants sat approximately 60 cm away from a 15 in. monitor and wore a set of headphones with a microphone attached (Plantronics Audio 326 PC Headset). Participants performed two picture naming, number naming, word-to-picture matching and number matching tasks per session (one prior to rTMS and one inside the rTMS induced refractory period—see above). The order of the tasks was counterbalanced across participants. Within a single experimental session participants saw all 80 picture naming and 80 picture matching stimuli, as well as all 40 number naming and 40 number matching stimuli. The experiment began with participants performing the four tasks with half of the stimuli prior to the application of TMS. The experimental trials were preceded by practice blocks of 10 trials per stimulus set.

*Naming:* For the naming tasks a fixation point appeared in the centre of the screen for 500 ms to signal the start of each trial. Stimuli were presented singly in the centre of the screen for a maximum of 2000 ms. The items were presented to each participant in a different random order. The task was to simply speak out loud the name of the object or number presented on the screen. The stimuli were presented until the response was given, with the response subsequently triggering a voice-key in the microphone and displaying a blank screen for an interval of 500 ms. The microphone recorded the participant’s response, with the computer recording the latency of each response via the DMDX Digital VOX software. Accuracy was determined offline by listening to the recordings.

*Matching:* As in the naming tasks the matching tasks began with a fixation point to signal the start of the trial. Participants heard through the headphones the name of a picture or a six-digit number, at the end of which two choice stimuli were immediately presented in the centre of the screen. Participants were required to select the picture or number which matched the spoken name that they had heard in the headphones. They did this by pressing the ‘shift’ key corresponding to the image on the screen (i.e. they pressed the left hand shift key to indicate the image on the left hand side of the screen). The stimuli were presented for a maximum of 2000 ms and were presented in a random order. The computer recorded the accuracy of the participant’s responses.

Pobric et al. (2007) noted that semantic decision times were suppressed for approximately 20 min after 10 min of 1-Hz rTMS, hence this was the duration used in this study. After 10 min of rTMS stimulation participants performed the four tasks again with the remaining sets of stimuli (the post-TMS condition). TMS was applied to either the left or right anterior temporal lobe in the first session, with TMS applied to the participant’s other anterior temporal lobe after a period of at least 2 weeks (to prevent practice effects). The order of temporal lobe stimulated on the first session was counterbalanced between participants to prevent order effects.

### Anatomical MRI acquisition

High resolution T1-weighted 3D anatomical images were acquired for all participants using a 3T Philips MR Achieva scanner (Philips Electronics, The Netherlands). MRI scanning parameters included an in-plane resolution of 1 mm and a slice thickness of 1.8 mm. An acquisition matrix of 256 × 256 voxels was used, however, the number of adjacent axial slices acquired for each participant varied to a maximum of 240, depending on the size of the participant’s head. This is because full head scans were required for accurate co-registration of the MRI images to the participant’s head. The high resolution T1-weighted images enabled the observation of the fine individual cortex folding, which was used as anatomical landmarks for the TMS targets.

### Selection of TMS site

The participant’s scalp was co-registered with the structural T1-weighted MRI scans using both MRIreg (http://www.mricro.com/mrireg.html) and an Ascension Minibird magnetic tracking system (http://www.ascension-tech.com). Prior to the administration of TMS a series of scalp landmarks were identified for co-registration with the MRI image and Minibird coordinates (nasion, tip of nose, chin, vertex, left/right tragus, left/right top of ear, left/right ear canal). Post-calibration the method of least squares linear regression was utilized to align the two frames of reference (overlaying the T1-weighted MRI image with the location of the participant’s head in 3D space). This allowed the comparison of the position of the Minibird on the scalp to the position of the underlying cortex. The anatomical landmark for the anterior temporal lobes in each participant was identified by measuring 10 mm posterior from the tip of the temporal pole, along the middle temporal gyrus. This site has been used in previous rTMS studies probing the semantic function of the left and right ATLs (Lambon Ralph et al. 2009; Pobric et al. 2010a). Our working definition of the ATL includes the anterior portion of all five temporal gyri. Recent evidence indicates that all five gyri are involved in semantic representation, though with a gradient of specialization, whereby the superior and middle temporal gyri are most strongly implicated in auditory-verbal knowledge and the fusiform and parahippocampal gyri more specialised for visual semantic knowledge (for review, see Lambon Ralph et al. (2016)). By selecting a site in the middle temporal gyrus, we aimed to target the portion of the ATL likely to be closely involved in naming. The site is also on the lateral surface of the temporal lobe, and therefore, closer to the scalp and in a suitable location to administer rTMS.

Once this location had been identified for both anterior temporal lobes in each participant, one lobe was selected for testing and the scalp location immediately above the appropriate temporal lobe was marked with a permanent marker. Across all participants, the mean left MNI coordinates for the anterior temporal lobe were (−53, 4, −32) in standard space, with the mean right MNI coordinates for the anterior temporal lobe being (52, 2, −28) in standard space.

### Stimulation parameters

Stimulation was provided by a MagStim Rapid2 stimulator (Magstim Co., Whitland, UK) with the assistance of two external boosters (maximum output approximately 2.2 T). A 70-mm figure-of-eight coil was utilized to apply the magnetic stimulation. Each testing session began with the determination of the individual motor threshold for each individual participant. This was identified as a visible twitch in the relaxed contralateral abductor pollicis brevis muscle. Stimulation was set at 120% of motor threshold for each participant, corresponding to an average stimulation intensity of 61% ± 5.52 (mean ± SD) of stimulator output. Repetitive pulse TMS was then applied at 1 Hz for 10 min (600 s) to either the left or right anterior temporal lobe. The coil was held secure over the identified stimulation site at such an orientation that the maximum induced current flowed approximately in the anterolateral direction along the middle temporal gyrus. However, compromises were reached with participants due to the uncomfortable nature of anterior temporal lobe stimulation (i.e. the inducement of facial and neck muscle contractions) Thus, in light of the knowledge that manipulating the orientation of the coil can minimize discomfort, changes in orientation were sometimes enacted where necessary.

## Results

Data from one participant were excluded from all analyses due to problems with voice-key insensitivity in one session yielding inaccurate response times. Any trials in which the microphone was inadvertently activated (less than 1% of trials), or to which the participant gave an incorrect response, were excluded from the reaction time analysis. Repeated measures ANOVAs were conducted on the reaction time and accuracy data. Pre-planned comparisons were conducted on the pre and post TMS values to determine the significance of the stimulation effect in each condition.

**Fig. 1:**
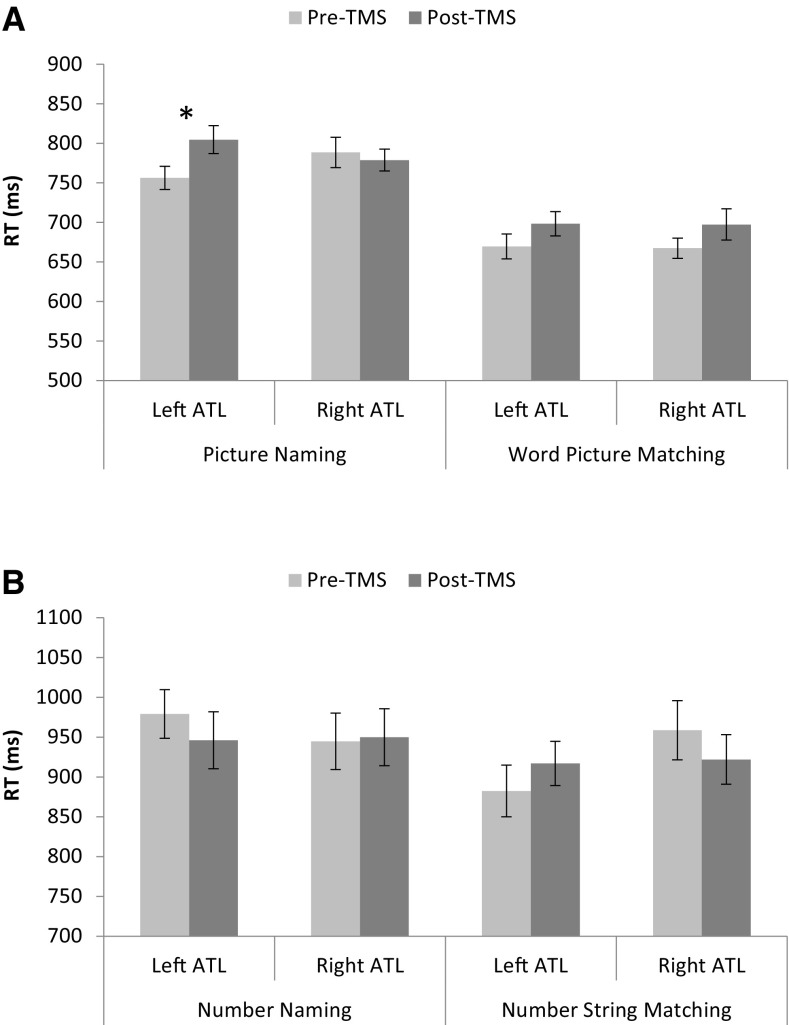
**a** *Top panel* shows mean reaction times for the picture naming and spoken word-to-picture matching tasks according to laterality and TMS. **b** *Bottom panel* shows mean reaction times for the number naming and spoken number-to-string matching tasks according to laterality and TMS. *Error bars* are standard error (across participants). *Asterisks* represent a significant effect of TMS, *p* < .005

Reaction time data, shown in Fig. 1, was analysed using a 2 (modality: semantic vs. numeric) × 2 (task: naming vs matching) × 2 (stimulation: pre-TMS vs. post-TMS) × 2 (laterality: left ATL vs. right ATL) repeated measures ANOVA. The results revealed a main effect of task such that naming responses were slower than matching decisions [*F*(1,10) = 15.65, *p* = .003; $\eta_{P}^{2}$ = 0.610]. There was also a significant four-way interaction [*F*(1,10) = 5.64, *p* = .039; $\eta_{P}^{2}$ = 0.360]. Further analyses of the picture data using a 2 (task: naming vs matching) × 2 (stimulation: pre-TMS vs. post-TMS) × 2 (laterality: left ATL vs. right ATL) repeated measures ANOVA again revealed a main effect of task [*F*(1,10) = 9.87, *p* = .012; $\eta_{P}^{2}$ = 0.481], and a significant three-way interaction [*F*(1,10) = 6.46, *p* = .029; $\eta_{P}^{2}$ = 0.392]. Analyses of the picture naming data using a 2 (stimulation: pre-TMS vs. post-TMS) × 2 (laterality: left ATL vs. right ATL) repeated measures ANOVA revealed a marginally significant interaction between stimulation and laterality [*F*(1,10) = 3.93, *p* = .076; $\eta_{P}^{2}$ = 0.282], and pre-planned comparisons revealed a significant inhibitory effect of TMS on picture naming latency when applied to the left ATL [*t*(10) = 3.37, *p* = .007], but not the right ATL [*t*(10) = .47, *p* = .651]. Analyses of the matching data using a 2 (stimulation: pre-TMS vs. post-TMS) × 2 (laterality: left ATL vs. right ATL) repeated measures ANOVA revealed no significant effects, nor did the pre-planned comparisons reveal significant stimulation effects.

In contrast to the picture data, analyses of the number data using a 2 (task: naming vs matching) × 2 (stimulation: pre-TMS vs. post-TMS) × 2 (laterality: left ATL vs. right ATL) repeated measures ANOVA revealed no significant effects, nor did the pre-planned comparisons reveal significant stimulation effects for any condition.

**Fig. 2:**
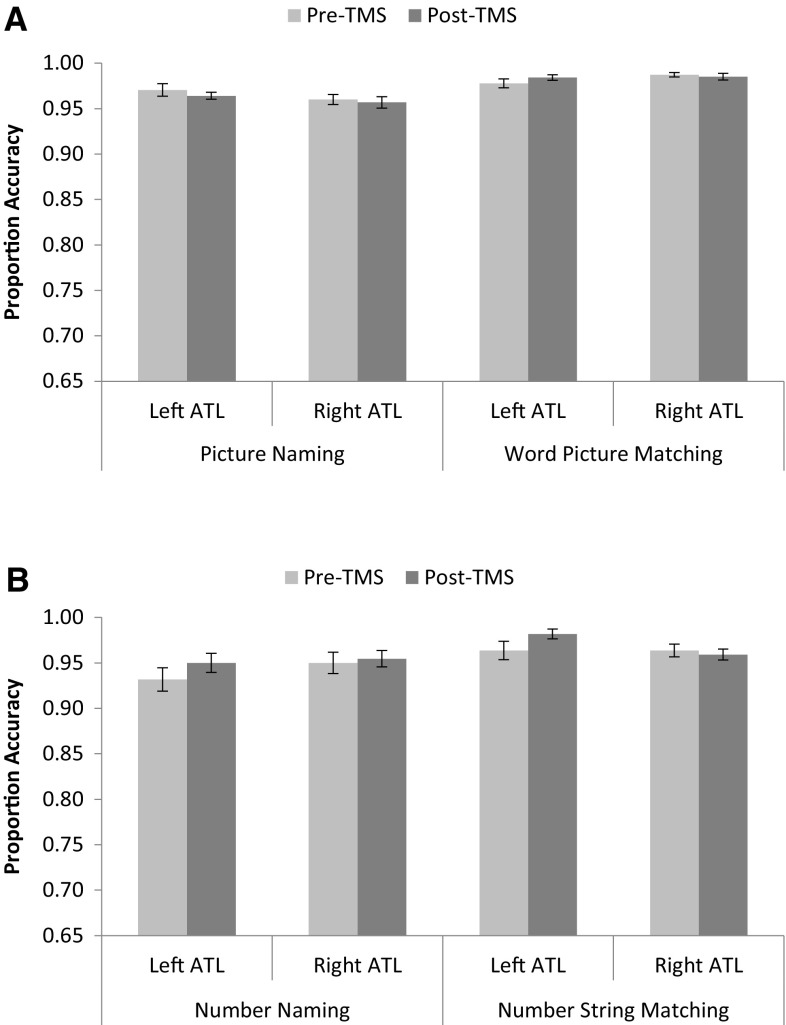
**a** *Top panel* shows mean accuracy for the picture naming and spoken word-to-picture matching tasks according to laterality and TMS. **b** *Bottom panel* shows mean accuracy for the number naming and spoken number-to-string matching tasks according to laterality and TMS. *Error bars* are standard error (across participants)

A parallel 2 (modality: semantic vs. numeric) × 2 (task: naming vs matching) × 2 (stimulation: pre-TMS vs. post-TMS) × 2 (laterality: left ATL vs. right ATL) repeated measures ANOVA on the accuracy data, shown in Fig. 2, revealed only a significant main effect of task such that naming responses were less accurate than matching decisions [*F*(1,10) = 12.54, *p* = .005; $\eta_{P}^{2}$ = 0.556].

## Discussion

This study provides the first investigation of the impact of laterality of a rTMS induced virtual lesion of the anterior temporal lobes on picture naming, a semantic task that also involves speech production. We found that TMS produced a significant decrement in naming performance when applied to the left but not the right ATL, with a marginally significant interaction between laterality and stimulation for picture naming indicating that the disruptive effect of TMS was greater when delivered to the left than the right ATL. To control for the contribution of left-lateralised speech production processes, we also considered the effect of left and right ATL stimulation on number naming. We found no performance decrement associated with TMS in this task, which indicates that the impact of left ATL TMS on picture naming cannot be attributed to the disruption of proximal left-lateralised inferior frontal regions associated with speech output.

Our naming results concord with previous demonstrations that picture naming is disrupted by left ATL TMS, but we have established for the first time that this effect is specific to the left ATL, with no such decrement apparent after right ATL stimulation. According to the connectivity view of ATL specialization (Lambon Ralph et al. 2001; Schapiro et al. 2013; Rice et al. 2015a), the left ATL is more connected to left-lateralised speech production areas than the right ATL, and hence it plays a greater role in semantic tasks involving phonological output. Our results strongly support this hypothesis. Picture naming involves recognition of the depicted object and activation of the name’s phonology. We interpret our results as showing that when the left ATL is stimulated, this disrupts the latter process, reducing the activation to inferior frontal regions associated with semantically driven speech production (Smith et al. 2001). When the right ATL is stimulated, it appears that the left ATL is capable of managing both of these processes (at least at the level of difficulty of items in this study). The ATLs are structurally connected via the anterior commissure (Catani and Thiebaut de Schotten 2008) and have high intrinsic connectivity that is increased in the semantic task of synonym judgement (Binney and Lambon Ralph 2015; Jung and Lambon Ralph 2016). Online upregulation of the contralateral ATL after TMS during synonym judgement has been seen in functional imaging studies (Binney and Lambon Ralph 2015; Jung and Lambon Ralph 2016). Our results demonstrate that even increased reliance on the right ATL after left ATL stimulation is not sufficient to maintain normal speech production, consistent with the performance of SD patients with strongly left-lateralised ATL pathology (Graham et al. 1995).

We also contrasted picture naming performance with a version of a task often used to assess receptive semantic knowledge in SD, namely spoken word-to-picture matching, and we found no reliable negative impact of TMS irrespective of laterality (and no effect of TMS on the control task of spoken-written number matching). This failure to find a significant effect runs counter to previous reports of significant bilateral ATL rTMS disruption using written synonym judgement and word and picture semantic association tasks (Lambon Ralph et al. 2009; Binney and Lambon Ralph 2015; Pobric et al. 2010a). It also runs counter to the SD literature showing speech comprehension deficits with this kind of task, however, it should be noted that spoken word-to-picture matching performance is often preserved relative to naming (Lambon Ralph et al. 2001). In essence, spoken word-to-picture matching is an easier task than picture naming, and indeed reaction times were lowest and accuracy was highest for this task. A receptive task like matching where one must prepare a simple motor response to select from a small number of alternatives after hearing the name would be expected to be faster than an expressive task like naming where one must prepare a complex speech response selected from a very large number of alternatives with response times also including picture decoding.

Spoken word-to-picture matching involves the recognition of a spoken name and matching the associated semantic activation to a picture. Given that we know from the naming results that the left ATL is capable of managing picture recognition and speech output after right ATL stimulation, then it is not surprising that right ATL stimulation did not affect spoken word-to-picture matching performance. The fact that left ATL stimulation did not affect spoken word-to-picture matching but did significantly impair picture naming performance demonstrates that the left ATL plays a particular role in the generation of phonological output, rather than phonological processing more generally. The absence of any detrimental effect of TMS on spoken word-to-picture matching suggests that this relatively easy task can be well supported by each anterior temporal lobe independently, consistent with the later decline of spoken word–picture matching than naming performance in semantic dementia, as atrophy becomes increasingly bilateral over time (Brambati et al. 2009; Rohrer et al. 2009). Although our results show a limited role for the right ATL in the picture naming and word-to-picture matching tasks we used, both left and right ATL stimulation do disrupt performance in harder semantic tasks like synonym judgement (Lambon Ralph et al. 2009) and semantic association judgement (Pobric et al. 2010a). It would seem, therefore, that the extra demands placed on the semantic system in these judgement tasks over and above simple cross-modality identity matching means that unilateral ATL activation is not sufficient to support normal performance.

Our experiment set out to directly test the connectivity account of ATL specialization (Lambon Ralph et al. 2001; Schapiro et al. 2013; Rice et al. 2015a), and related theories (Drane et al. 2013) which posit a particular role for the left ATL specifically in speech production. As picture and words were involved in both of our semantic tests, our study was not ideally designed to test the modality account of ATL specialization, which predicts material specificity such that the left ATL is more involved in verbal processing and the right ATL in non-verbal processing (Gainotti 2012). Within this view, we may have expected to see disruption of processing due to ATL stimulation in both tasks irrespective of laterality, as they both involve verbal and non-verbal processing (i.e. names and pictures). The argument could also be made within the modality account, however, that each ATL in isolation is sufficient to support adequate performance in these relatively easy tasks that focus on identification, especially if stimulation produces some degree of upregulation of the contralateral ATL. While this argument could explain the absence of effects, it does not explain the specific and significant disruptive effect of left ATL stimulation on picture naming. To account for our observed pattern of results then, the modality account of ATL specialization would have to incorporate an additional assumption that semantic tasks involving generation as opposed to comprehension of phonology load more heavily on verbal knowledge. In summary, the observed results confirm the key prediction of the connectivity account of ATL specialization, but could also be accommodated by the modality account. In fact these two accounts are not mutually exclusive, as the left ATL may be specialised for verbal processing in tandem with higher connectivity to left frontal speech production areas.

The connectivity account of ATL specialization (Lambon Ralph et al. 2001; Schapiro et al. 2013; Rice et al. 2015a), is supported by both functional and structural imaging data showing closer linkage between the ATL and left-lateralised frontal regions involved in speech output processing. Resting state functional connectivity between the anterior temporal lobe and inferior frontal gyrus is higher in the left than right hemisphere (Hurley et al. 2015). Structurally, both the uncinate fasciculus and the inferior frontal occipital fasiculus are key white matter tracts of the ventral meaning-based pathway (Bajada et al. 2015), with terminations in areas of the left inferior frontal gyrus. Both of these tracts in the left hemisphere have been linked to a behavioural semantic factor with a high loading on picture naming in healthy older adults (De Zubicaray et al. 2011) and across three semantic tasks including naming in a sample of brain damaged patients (Han et al. 2013). Consistent with the connectivity account of ATL specialization, the volume of the uncinate has been found to be larger in the left than the right hemisphere in healthy adults (Leng et al. 2016; Parker et al. 2005). More recently, higher connectivity in left than right uncinate has been reported specifically for the volume of the dorsolateral component (Hau et al. 2016). Intracranial electrical stimulation studies, however, have shown a role for the left inferior frontal occipital fasiculus rather than the left uncinate in picture naming (Duffau et al. 2009) leading to the proposal that the left uncinate forms part of an indirect and compensatable ventral language pathway (Duffau et al. 2013). Yet naming deficits are observed after removal of the left uncinate (Papagno et al. 2011), it is specifically the left uncinate that is disrupted in semantic dementia (Iaccarino et al. 2015), and the integrity of the left uncinate has recently been linked specifically with speech production capacity in chronic aphasia (Ivanova et al. 2016), consistent with the current findings.

Our results also bear on specialization of subregions within the left anterior temporal lobe. We stimulated the lateral anterior temporal lobe (−53, 4, −32) as this is amenable to TMS, and the resultant disruption of naming performance suggests that this region is involved in the linkage of semantics with phonological output. This is consistent with recent functional imaging work on reading (Hoffman et al. 2015), where it was specifically this particular area of the left lateral ATL that showed higher activation for (a) irregular words and (b) participants with a stronger degree of semantic reliance for irregular word reading. In addition, a recent investigation of determining the relatedness of pictured objects and double object picture naming found an area of the left lateral ATL (−51, 9, −24) that was particularly associated with retrieval of a specific concept for picture naming (Sanjuán et al. 2015). It seems that our study adds to an emerging body of evidence that it is the lateral portions of the left ATL that are especially involved in activation of phonological forms, which provides additional information concerning the mechanism underpinning the specialization proposed in the connectivity account.

Our study used virtual lesion rTMS to provide the first evidence that disruption of naming from ATL stimulation is seen only for the left, with no comparable decrement on the right. This disruption could not be attributed to interference from TMS to nearby inferior frontal areas involved in preparation for speech production, as there was no effect of stimulation on a number naming control task. This result was predicted according to the connectivity view of ATL specialization, which is also supported by recent structural and functional imaging data showing a leftward bias in the links between the anterior temporal lobe and inferior frontal regions. Our results are in line with recent evidence suggesting specialization within the left anterior temporal lobe such that it is the lateral regions that provide the specific semantic activation needed to drive speech production. Future neurostimulation and functional imaging studies could explore whether similar effects are observed in tasks that involve activation of phonological forms without any requirement for overt articulation.

## Appendix

**Table:**

Picture naming	Word picture matching	Number naming	Number string matching
Ant	Basket	975,461	360,128
Backpack	Bell	438,729	152,207
Beard	Brush	759,138	682,821
Bone	Butter	469,170	402,479
Camera	Cannon	739,610	392,462
Cat	Chimney	138,076	266,203
Chair	Closet	569,421	170,873
Church	Crown	259,081	359,404
Cigarette	Dragon	569,048	500,751
Cross	Elephant	780,254	408,263
Dog	Flashlight	230,476	551,016
Door	Glove	352,691	919,786
Dress	Jar	860,453	712,268
Finger	Key	861,349	883,457
Flower	Knife	524,871	920,299
Fountain	Lamp	261,093	852,518
Glasses	Mouse	419,635	718,137
Goat	Mushroom	935,648	796,424
Hair	Pants	368,471	909,495
Hanger	Pencil	295,473	508,038
Heart	Priest		
Horn	Pumpkin		
Iron	Pyramid		
King	Rope		
Ladder	Sandwich		
Map	Screwdriver		
Moon	Shovel		
Owl	Snake		
Piggybank	Tent		
Plug	Tiger		
Radio	Toaster		
Refrigerator	Toilet		
Rocket	Tractor		
Roof	Truck		
Table	Turkey		
Thermos	Volcano		
Thumb	Watermelon		
Typewriter	Wheelchair		
Umbrella	Wing		
Vase	Wrench		
Accordion	Bat	395,647	541,102
Arrow	Bowl	964,378	489,015
Bed	Cage	879,546	199,651
Belt	Can	705,814	318,742
Binoculars	Cane	572,689	797,500
Bride	Cheese	982,153	277,745
Bridge	Cherry	287,503	304,966
Car	Clock	243,569	466,475
Corn	Clown	381,427	364,384
Dentist	Eskimo	836,120	317,958
Ear	Fox	876,352	768,795
Fan	Genie	689,147	802,893
Fence	Guitar	423,156	660,820
Fish	Helicopter	462,153	746,875
Globe	Helmet	765,149	633,790
Heel	Leaf	532,769	619135
Hook	Nail	314,879	589,461
Ironing board	Necklace	914,576	903,442
Jacket	Needle	352,096	648,575
Kite	Peacock	607,429	573,109
Lawnmower	Piano		
Match	Pig		
Mirror	Pirate		
Orange	Popcorn		
Pipe	Potato		
Puzzle	Sailor		
Queen	Scorpion		
Rain	Skunk		
Rainbow	Sled		
Road	Spaghetti		
Rock	Spatula		
Sewing machine	Statue		
Shark	Sword		
Shoe	Tie		
Slide	Tire		
Spider	Turtle		
Sun	Vest		
Tree	Whale		
Tweezers	Witch		
Window	Zipper		
